# Potential Application of Leelamine as a Novel Regulator of Chemokine-Induced Epithelial-to-Mesenchymal Transition in Breast Cancer Cells

**DOI:** 10.3390/ijms23179848

**Published:** 2022-08-30

**Authors:** Young Yun Jung, Jae-Young Um, Gautam Sethi, Kwang Seok Ahn

**Affiliations:** 1 Department of Science in Korean Medicine, Kyung Hee University, 24 Kyungheedae-ro, Dongdaemun-gu, Seoul 02447, Korea; 2 Department of Pharmacology, Yong Loo Lin School of Medicine, National University of Singapore, Singapore 117600, Singapore; 3 NUS Centre for Cancer Research (N2CR), Yong Loo Lin School of Medicine, National University of Singapore, Singapore 117599, Singapore

**Keywords:** leelamine, CXCR7, CXCR4, EMT, MnSOD, breast cancer

## Abstract

CXCR7 and CXCR4 are G protein-coupled receptors (GPCRs) that can be stimulated by CXCL12 in various human cancers. CXCR7/4–CXCL12 binding can initiate activation of multiple pathways including JAK/STAT and manganese superoxide dismutase (MnSOD) signaling, and initiate epithelial–mesenchymal transition (EMT) process. It is established that cancer cell invasion and migration are caused because of these events. In particular, the EMT process is an important process that can determine the prognosis for cancer. Since the antitumor effect of leelamine (LEE) has been reported in various previous studies, here, we have evaluated the influence of LEE on the CXCR7/4 signaling axis and EMT processes. We first found that LEE suppressed expression of CXCR7 and CXCR4 both at the protein and mRNA levels, and showed inhibitory effects on these chemokines even after stimulation by CXCL12 ligand. In addition, LEE also reduced the level of MnSOD and inhibited the EMT process to attenuate the invasion and migration of breast cancer cells. In addition, phosphorylation of the JAK/STAT pathway, which acts down-stream of these chemokines, was also abrogated by LEE. It was also confirmed that LEE can induce an imbalance of GSH/GSSG and increases ROS, thereby resulting in antitumor activity. Thus, we establish that targeting CXCR7/4 in breast cancer cells can not only inhibit the invasion and migration of cancer cells but also can affect JAK/STAT, EMT process, and production of ROS. Overall, the findings suggest that LEE can function as a novel agent affecting the breast cancer.

## 1. Introduction

**Figure 1:**
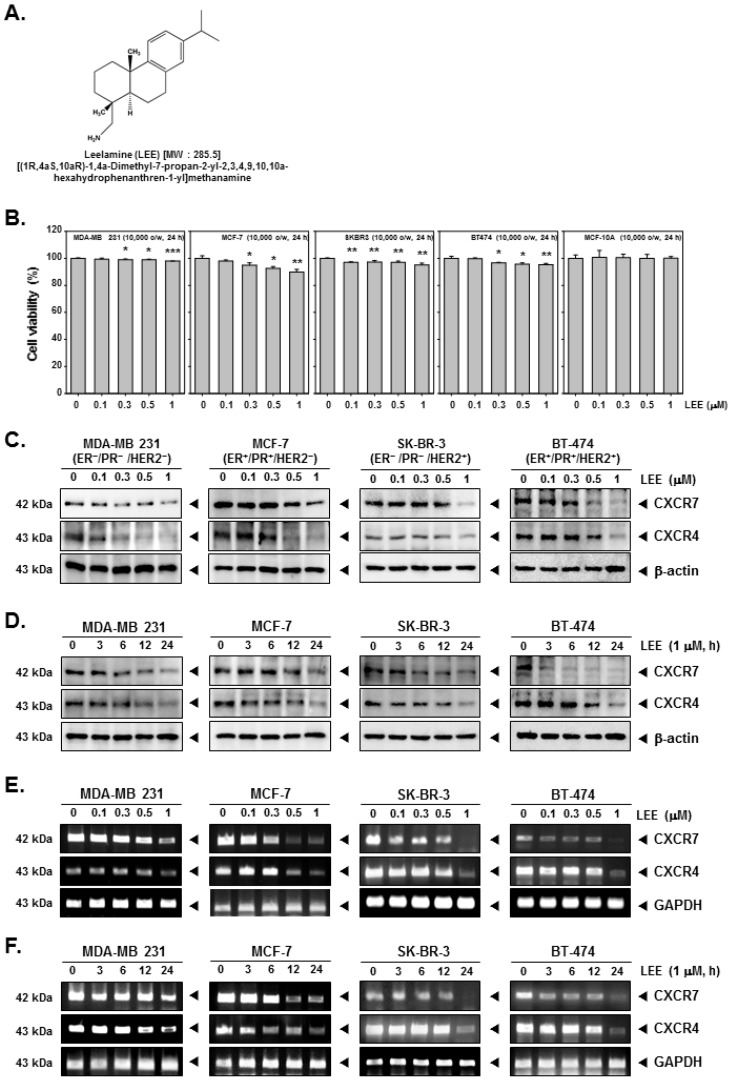
Effects of LEE on inhibition of CXCR7 and CXCR4 in human breast cancer cells. (**A**) The chemical structure of LEE. (**B**) Human breast cancer MDA-MB231, MCF-7, SK-BR-3, and BT-474 cells were treated by LEE with indicated concentrations for 24 h. Then the cell viability was measured by MTT assay. (**C**,**D**) The cells were treated with LEE at indicated concentrations and for indicated time periods. The protein expression levels of CXCR7/4 were analyzed by Western blot analysis. (**E**,**F**) mRNA levels of LEE-treated breast cancer cells were analyzed by RT- PCR. All the experiments were individually repeated at least thrice. *** *p* < 0.001 vs. non-treated (NT) cells, ** *p* < 0.01 vs. non-treated (NT) cells, and * *p* < 0.05 vs. non-treated (NT) cells.

Leelamine (LEE, dehydroabietylamine, Figure 1) is a lysosomotropic compound containing lipophilic diterpene amine that can be extracted from pine’s bark trees naturally [1,2]. The potential anticancer effects of LEE have been reported in various previous studies. In a recent study, we have reported that LEE induced both autophagy and apoptosis with modulation of the STAT5 pathway in myelogenous leukemia cells [3]. The ability of LEE to inhibit the STAT3/JAK signaling pathway with activation of PTPε in multiple myeloma cells has also been reported [4]. Moreover, induction of the release of cytochrome c to cytosol and activation of Bax and Bak by LEE selectively killed breast cancer [5]. It has also been found that the production of reactive oxygen species (ROS) was increased upon exposure to LEE [5]. In addition, inhibition of androgen receptors, which are potential targets of androgen-receptor-positive cancer cells, by LEE has been reported [6]. In prostate cancer cells, LEE caused inhibition of fatty acid synthesis mediated by sterol regulatory element-binding protein 1 (SREBP1) [7]. In another study, it was reported that the anticancer effects of LEE in the prostate cancer were mediated through downregulation of cMyc in vitro and in vivo [8]. In the present study, the anticancer activity of LEE in human breast cancer cells was explored.

Chemokine receptors 7 (CXCR7) and 4 (CXCR4) are G protein-coupled receptors (GPCRs) that can be stimulated by a ligand, CXCL12, in various human cancers [9,10,11]. CXCR7 has been known to play a role in the central nervous system [9], neurogenesis [12], cardiogenesis [13], and angiogenesis [14]. CXCR4 is also a GPCR that is encoded on chromosome 2.1 with seven-spanning transmembrane domains (352 amino acids, 48 kDa) [15,16]. CXCL12–CXCR4 binding can initiate multiple signaling pathways to regulate intracellular calcium flux, chemotaxis, transcription, and cell survival [17]. When CXCL12 binds to CXCR4, intracellular calcium movement is promoted, the receptor is ubiquitinated, then endocytosis and lysosomal degradation occur [18]. In cortical astrocytes and Schwann cells, in which CXCR4 is also expressed, CXCR7 can control CXCL12 signaling [19]. A number of previous studies have demonstrated that CXCR7 can internalize CXCL12 to trigger intracellular pathways such as MAPK, JAK/STAT3, and Akt via forming heterodimers with CXCR4 [20,21,22,23]. The CXCL12-stimulated CXCR7/4 complex can activate downstream cell signals including MAPKs, which can, in turn, induce cellular migration and invasion [21,24,25]. Overall, the CXCR7 signaling pathway is more dependent on the cellular context and on relative expression compared with CXCR4 [26]. Thus, targeting CXCR7/4 can be a useful strategy to inhibit the metastasis of cancer cells.

Manganese superoxide dismutase (MnSOD) is manganese-dependent mitochondrial enzyme that detoxifies reactive oxygen species (particularly converts superoxide (O_2_^−^) to hydrogen peroxide (H_2_O_2_)) to maintain the homeostatic condition of the cells [27,28,29]. In previous studies, it has been reported that the regulation of MnSOD acts as a switch in the epithelial–mesenchymal transition (EMT) process through the regulation of cellular redox [30]. A particularly noteworthy finding is that it blocks the EMT process in colorectal cancer by reducing the level of MnSOD through overexpression of miR-212, a miRNA that can directly target MnSOD mRNA [31]. This report demonstrated that MnSOD played an essential role in the reduction of epithelial markers and elevation of mesenchymal markers in colorectal cancer cells [31]. In addition, in another study, inhibition of MnSOD was found to suppress various EMT-related transcription factors and MMPs in head and neck squamous cell carcinoma cells [32]. Matrix metalloproteinase (MMP) family proteins such as MMP-9 and MMP-2 play an important role in cancer progression by regulating invasion, proliferation, and metastasis [33,34,35,36,37]. 

The EMT process, primarily trigged by epithelial cells conversion to mesenchymal cells, can facilitate the induction of cellular invasion and migration [38,39,40,41,42,43]. The EMT process is an important physiological process that can regulated the cells metastasis, invasion, and migration [40,43,44,45,46]. The EMT process is mediated through various transcription factors, inflammatory cytokines, growth factors, and other proteins and enzymes [44]. It has been also reported that CXCL12 can induce the EMT process through modulating the CXCR4/7 axis to promote cellular invasion and migration [47,48].

In our study, we evaluated the anticancer potential of LEE to modulate the metastasis of breast cancer cells. As a result, it was observed that LEE inhibited CXCR7 as well as CXCR4 expression and the EMT process, and these were abrogated to same extent after stimulation with the ligand CXCL12. In addition, CXCR7 and CXCR4 levels were increased after MnSOD overexpression, and the anticancer efficacy of LEE was verified by inhibition of the various downstream signals including the EMT process and JAK/STAT. Our study suggested that LEE could abrogate the progression of breast cancer through affecting multiple signaling pathways.

## 2. Results

### 2.1. LEE Inhibited the Growth of Human Breast Cancer Cells and Affected Expression of CXCR7 and CXCR4

First, we confirmed the cell viability of LEE-treated human breast cancer MDA-MB231, MCF-7, SK-BR-3, and BT-474 cells and normal MCF-10A cells. The cells were treated with LEE (0, 0.1, 0.3, 0.5, and 1 μM) for 24 h and analyzed by MTT assay. It was found that at all the tested concentrations cell viability was above 90%, and no significant decrease was noted even in the viability of MCF-10A cells (Figure 1B and Figure S1). To investigate the effects of LEE on the EMT process, we selected the concentrations that exhibited the least toxicity. Thereafter, the expression level of CXCR7 and CXCR4 was evaluated at different concentrations and timepoints (Figure 1C,D and Figure S2). As shown in the results, the levels of both CXCR7 and CXCR4 were observed to decrease with increasing concentration (24 h) and time intervals. Under similar conditions, mRNA levels of CXCR7 and CXCR4 were also substantially decreased (Figure 1E,F and Figure S3). Overall, the results indicate that LEE exhibits significant inhibitory effects on CXCR7 and CXCR4 expression even at the lower concentrations.

### 2.2. LEE Abrogated the Migration and Invasion in Human Breast Cancer Cells

**Figure 2:**
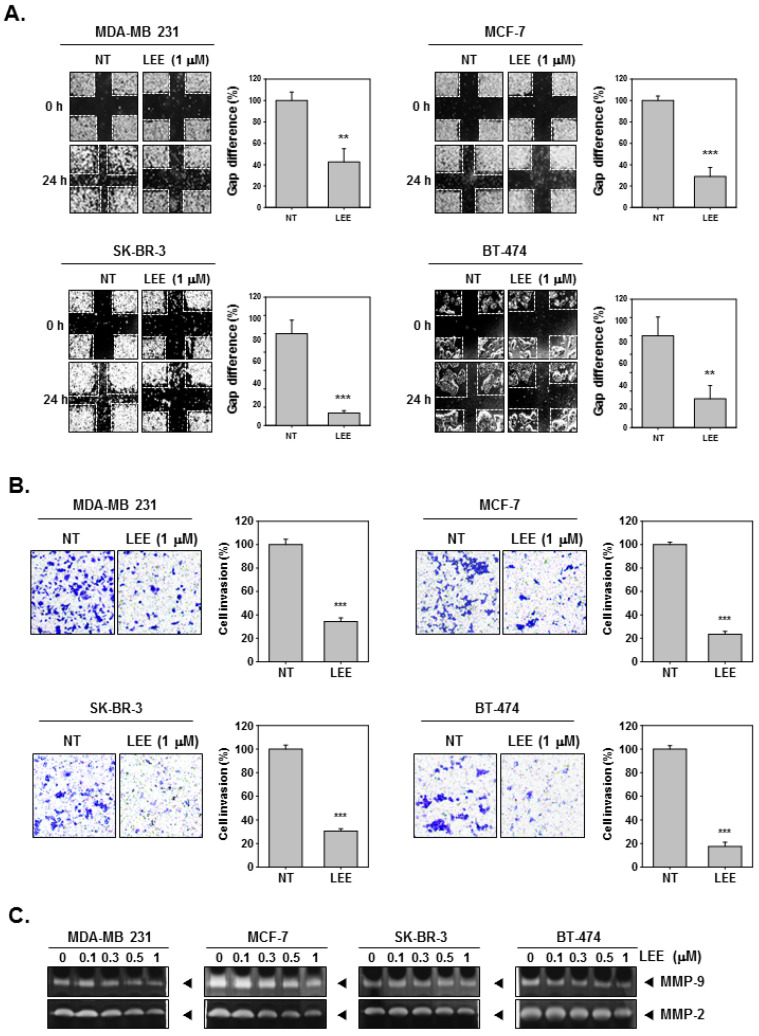
Inhibitory effects of LEE on cell invasion and migration. (**A**) Cell migration was evaluated by wound healing assay. (**B**) Cell invasion was observed by Boyden chamber assay. (**C**) MMP-9/2 activity was evaluated by zymography assay. Supernatants were obtained, concentrated, and analyzed. All the experiments were individually repeated at least thrice. *** *p* < 0.001 vs. non-treated (NT) cells, and ** *p* < 0.01 vs. non-treated (NT) cells.

Next, we investigated whether LEE exerted inhibitory effect on cellular migration and invasion activities. It was noted that the treatment of LEE could significantly suppress the cell migration of MDA-MB231, MCF-7, SK-BR-3, and BT-474 cells (Figure 2A). The gap difference was analyzed and compared as a percentage by measuring the width of wound and calculating how this changed relative to each non-treated (NT) and LEE treatment. NT cells showed growth by invasion of the wound over 24 h, however, LEE-treated cells did not relatively invade wounds at the same time. Then, we confirmed the cell invasion by Boyden chamber assay. The cells were treated with LEE (1 μM), then incubated in the upper chamber above the membrane for 6 h. During incubation for 6 h, cells could undergo invasion and migrate through the Matrigel membrane into the lower chamber. However, LEE inhibited invasion into the lower chamber by human breast cancer cells (Figure 2B). In addition, to investigate MMP-9 and MM-2 activation, we obtained the supernatants and concentrated them to prepare the sample. These samples were then loaded and separated on gelatin-containing SDS gels. Activation of MMP-9 and MMP-2 was observed to decrease with increasing concentrations of LEE (Figure 2C and Figure S4).

### 2.3. LEE Suppressed the EMT Process through Modulating EMT Markers

**Figure 3:**
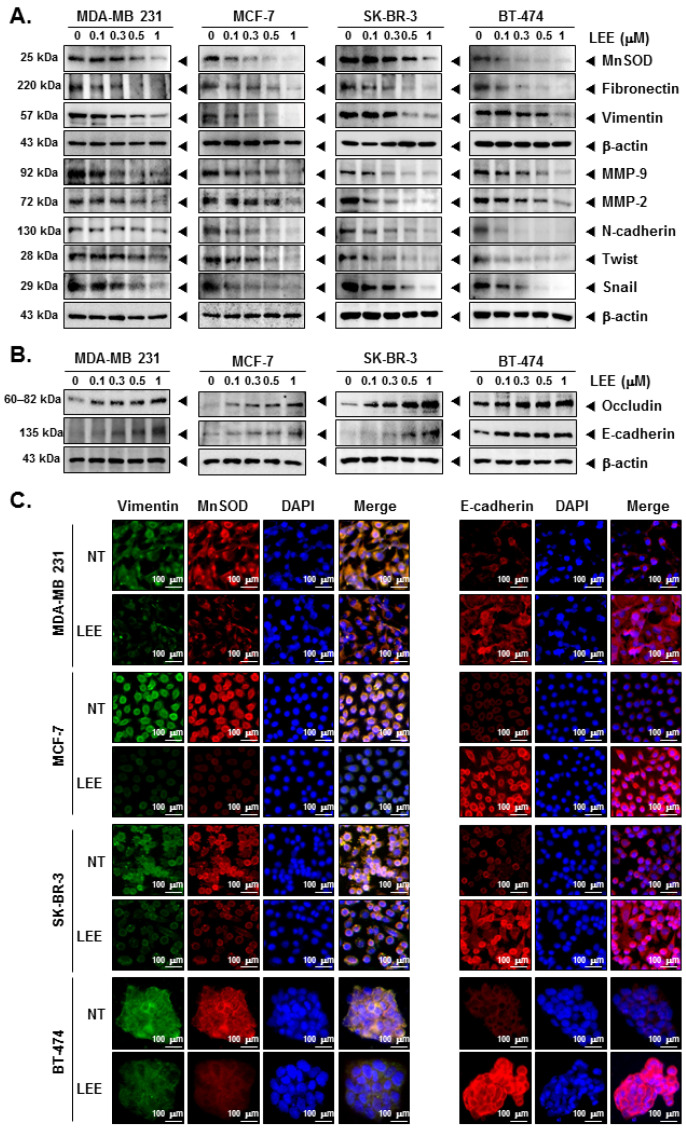
Regulation of EMT process by LEE in human breast cancer cells. (**A**,**B**) The expression of several EMT markers were analyzed by Western blot analysis in whole cell lysates. (**C**) Vimentin, MnSOD, and E-cadherin expression in the cells was observed by immunocytochemistry. DAPI was used to stain the nuclei in each cell. All the experiments were individually repeated at least thrice.

Next, we confirmed the effects of LEE in the EMT process to investigate the mechanisms through which LEE can affect cellular invasion and migration. The whole cell lysates were analyzed by Western blot analysis and results indicated that expression of MnSOD, MMP-9/2, and several mesenchymal markers, such as fibronectin, vimentin, N-cadherin, twist, and snail expression was decreased at various concentrations of LEE (Figure 3A and Figure S5). On the other hand, LEE increased the levels of different epithelial markers, such as occludin and E-cadherin (Figure 3B and Figure S6). Thereafter, vimentin, MnSOD, and E-cadherin expression in the cells was observed by immunocytochemistry. LEE suppressed the expression of vimentin and MnsOD, but E-cadherin level was induced by LEE (Figure 3C). Thus, LEE decreased the expression of mesenchymal markers and increased that of the epithelial markers to suppress the EMT process.

### 2.4. LEE Attenuated the EMT Process in CXCL12-Stimulated Breast Cancer Cells

**Figure 4:**
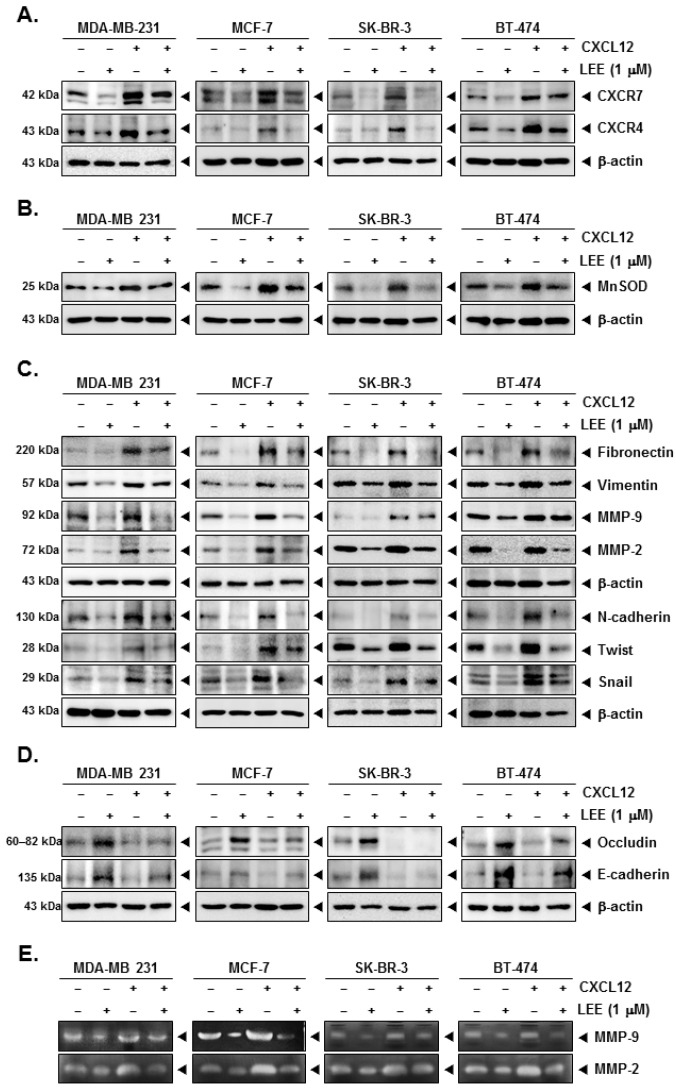
Effects of LEE on CXCR7/4 and several EMT markers in presence of CXCL12 stimulation. The cells were stimulated with CXCL12 (10 ng/mL) and treated with LEE (1 μM) for 24 h. Whole cell lysates were analyzed by Western blot analysis. The protein expression level of (**A**) CXCR7/4, (**B**) MnSOD, and (**C**,**D**) several EMT markers (fibronectin, vimentin, MMP-2/9, N-cadherin, twist, snail, occludin, E-cadherin) were probed using specific antibodies. (**E**) The supernatants were obtained and concentrated, and then activity of MMP-9/2 was evaluated by zymography. All the experiments were individually repeated at least thrice.

CXCL12 can stimulate and induce the expression of CXCR7 as well as CXCR4 and various EMT markers in human breast cancer cells. Despite the induction of CXCR7 and CXCR4 by CXCL12, LEE markedly decreased their expression (Figure 4A and Figure S7). Moreover, CXCL12 induced the levels of MnSOD and various mesenchymal markers, however, LEE suppressed these markers (Figure 4B,C, Figures S7 and S8). Although epithelial markers, such as occludin and E-cadherin, were inhibited by CXCL12, LEE treatment could also induce them effectively (Figure 4D and Figure S9). MMP-9 and MMP-2 levels were also induced by stimulation of CXCL12, and LEE substantially decreased their activation (Figure 4E and Figure S10).

### 2.5. LEE Reduced the Various Tumorigenesis Proteins in MnSOD-Overexpressing Breast Cancer Cells

**Figure 5:**
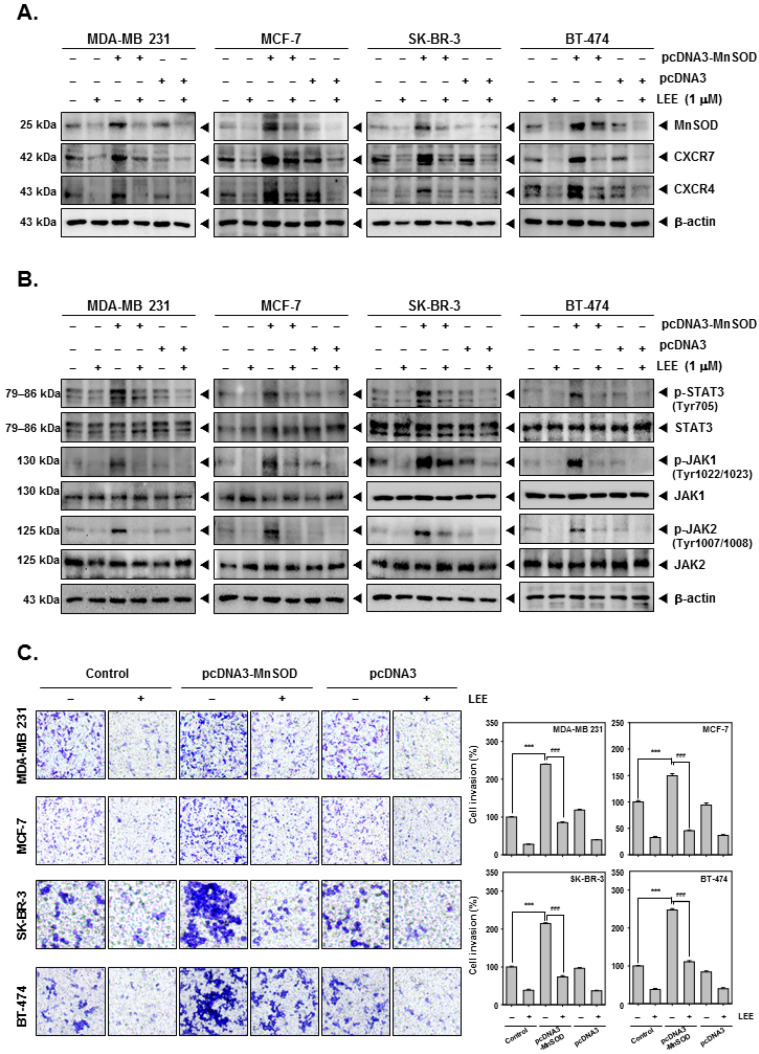
Inhibitory effects of LEE on MnSOD-overexpressing human breast cancer cells. The cells were transfected with pcDNA3-MnSOD, and thereafter treated with LEE. MnSOD-overexpressing cells were evaluated by Western blot analysis. The expression level of (**A**) MnSOD, CXCR7/4, (**B**) p-STAT3, STAT3, p-JAK1, JAK1, p-JAK2, and JAK2 were observed. (**C**) The cell invasion was evaluated by Boyden chamber assay using Nikon Eclipse Ts2 (magnification, 20×) (Nikon Instrument, Tokyo, Japan). All the experiments were individually repeated at least thrice. ### *p* < 0.001 vs. non-treated (NT) cells, and *** *p* < 0.001 vs. LEE treated cells.

To decipher the molecular mechanisms, MnSOD was overexpressed by pcDNA3-MnSOD transfection into breast cancer cells. It was found that both expression of CXCR7 and CXCR4 was increased by MnSOD overexpression. Despite MnSOD overexpression, LEE inhibited MnSOD, CXCR7, and CXCR4 levels (Figure 5A and Figure S11). In addition, analysis of the STAT3 signaling pathway also showed that phosphorylation of STAT3, JAK1, and JAK2 was substantially increased by MnSOD overexpression, but LEE inhibited activation by inhibiting their phosphorylation (Figure 5B and Figure S12). Moreover, the phosphorylation of STAT3, JAK1, and JAK2 was increased by MnSOD overexpression, but LEE inhibited their phosphorylation. Overexpression of MnSOD induced cell invasion, which was observed by Boyden chamber assay, and LEE treatment significantly reduced the invasive activity (Figure 5C).

### 2.6. LEE Induced ROS Production through Promoting GSH/GSSG Imbalance

**Figure 6:**
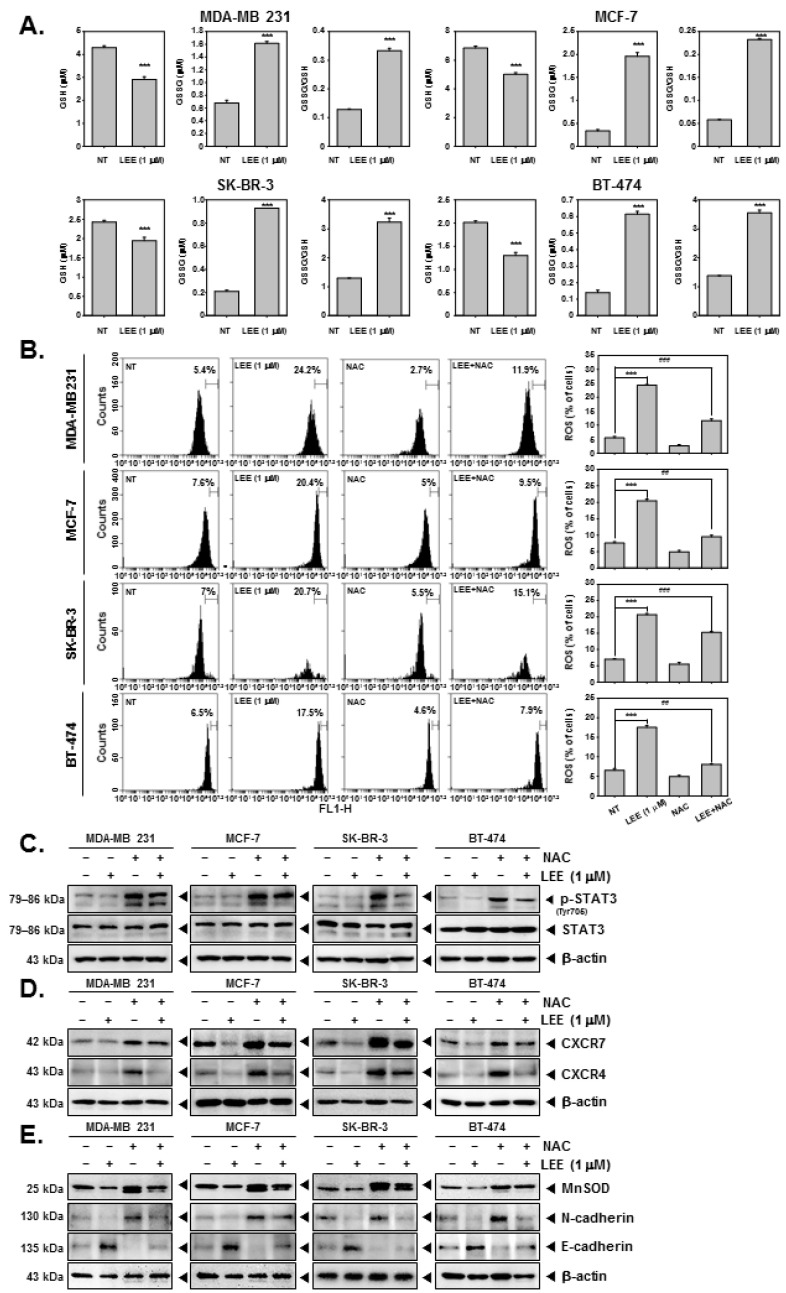
Induction of GSH/GSSG imbalance and ROS production by LEE. (**A**) The cells were treated with LEE, and GSH/GSSG was measured by GSH/GSSG assay. (**B**) The cells were pre-treated with LEE for 24 h, and stimulated by N-acetyl-l-cysteine (NAC) (3 mM) for 15 min. Then, ROS production was measured by flow cytometry. (**C**) Whole cell lysates were analyzed by Western blot analysis. Then, p-STAT3 and STAT expression levels were confirmed. (**D**,**E**) CXCR4/7 and EMT markers were evaluated by Western blot analysis. All the experiments were individually repeated at least thrice. ### *p* < 0.001 vs. LEE+NAC-treated cells, ## *p* < 0.01 vs. LEE + NAC-treated cells, and *** *p* < 0.001 vs. LEE-treated cells.

In human breast cancer MDA-MB 231, MCF-7, SK-BR-3, and BT-474 cells, LEE induced GSH/GSSG imbalance by reducing GSH while increasing GSSG level (Figure 6A). We also noted that ROS levels were increased upon exposure to LEE and it even maintained ROS production to some extent in N-acetyl-l-cysteine (NAC)-treated cells (Figure 6B). To further investigate the mechanism, we treated human breast cancer cells with both NAC and LEE. As shown in the results, NAC increased the activation of STAT3, CXCR7, and CXCR4, whereas LEE inhibited it. (Figure 6C,D, Figures S13 and S14). In addition, NAC induced MnSOD and N-cadherin levels whereas that of E-cadherin was reduced. However, LEE decreased MnSOD and N-cadherin expression and increased that of E-cadherin (Figure 6E and Figure S15). These results clearly demonstrate that LEE could increase ROS production through GSH/GSSG imbalance despite interference from NAC. 

## 3. Discussion

In this study, our goal was to investigate the diverse molecular mechanisms through which LEE could target the invasion and migration of breast cancer cells. We selected LEE as it has been previously reported to exhibit anticancer efficacy in some studies but its impact on the CXCR7/4 axis and EMT process has not been reported [3,4,5,6,7,8]. Thus, our study mainly examined the impact of LEE on the regulation of the EMT process by targeting the CXCR7/4 signaling axis in breast cancer cells. CXCR7/4 acts as a G protein-coupled receptor (GPCR) that can regulate intracellular calcium flux, chemotaxis, transcription, and cell survival [17]. As previously reported, CXCR7/4 can be stimulated by its ligand, CXCL12, which can then induce the JAK/STAT pathway and EMT processes [17,18,19,20,21,26]. In addition, the EMT process can also be regulated by MnSOD [30,31,32]. Our findings confirm that the antimetastatic activities of LEE were mediated primarily through the inhibition of CXCR7/4 and the EMT process in breast cancer cells. 

First, we verified the concentration that maintains cell viability above 90% through the MTT assay. The following experiments were carried out by selecting the specific concentration that exhibited the lowest cytotoxicity. Both CXCR7 and CXCR4 expression decreased as analyzed by Western blot and reverse-transcription PCR in a concentration- or time-dependent manner upon LEE treatment. In addition to the inhibition of CXCR7/4 expression by LEE, it was confirmed by wound healing and Boyden chamber assays that LEE could significantly suppress the invasion and migration of breast cancer cells. In addition, activity of MMPs, known to be related to the EMT process [32,33,34,35,36], was analyzed by zymography, and it was observed that LEE reduced the level of MMP-9/2 in breast cancer cell culture medium. These results suggested that LEE could consequently inhibit the invasion and migration of breast cancer cells and cause the suppression of MMP-9/2, which is involved in the EMT process. 

To further confirm the effect of LEE on the EMT process, whole cell lysates were analyzed by Western blotting to determine the expression of MnSOD [30,31], mesenchymal markers (Fibronectin, Vimentin, N-cadherin, twist, and snail) [49,50], MMP-9/2, and epithelial markers (occludin and E-cadherin) [51,52]. The results show a substantial decrease in mesenchymal markers along with a decrease in MnSOD level, and conversely, by observing increase in the levels of epithelial markers, it was demonstrated that LEE could regulate both cellular invasion and migration through modulating MnSOD and the EMT process. Thereafter, to confirm the association between CXCR7/4 and the EMT process, we checked the protein expression level under CXCL12 stimulation [47,48]. It was confirmed that treatment with CXCL12 increased the expression of MnSOD along with an increase in CXCR7/4. In addition, stimulation of CXCL12 induced the EMT process by promoting an increase in the levels of the various mesenchymal markers and a decrease in epithelial markers. Thereafter, it was noted that the level of MMP-9/2 in the culture medium, an extracellular environment, was also increased by CXCL12 treatment. However, despite CXCL12 treatment, LEE could still inhibit the EMT process through causing a decrease in MnSOD and mesenchymal markers with an increase in epithelial markers. Through these results, we were able to conclude that the modulation of the EMT process and CXCR7/4 axis contributes to the anticancer efficacy of LEE. 

Furthermore, to confirm the relationship between MnSOD and CXCR7/4, we overexpressed MnSOD in breast cancer cells. Interestingly, overexpression of MnSOD was observed to increase the level of CXCR7/4, and it was found that the protein expression level of the JAK/STAT pathway increased upon stimulation by CXCR7/4 [53,54]. In addition, overexpression of MnSOD induced invasion and migration of breast cancer cells, which was observed to be inhibited by LEE.

Finally, we evaluated the modulation of ROS production by LEE in tumor cells. ROS can be generated by mitochondria and other cellular elements, and exogenously by exposure to radiation, drugs, tobacco, pollutants, smoke, and xenobiotics [55]. ROS can regulate cell proliferation, survival, invasion, angiogenesis, and inflammation affecting multiple signaling pathways such as STAT3, NF-κB, and other proteins and enzymes [55]. Moreover, in cancer cells it has been reported that increased ROS levels can induce cancer cell death through anticancer signaling [56,57]. Imbalance between glutathione (GSH) and oxidized GSH (GSSG) can cause the ROS [58,59]. Reduction of GSSG in response to increased GSH can induce ROS levels, which in turn can activate antitumor signaling mechanisms [58,59]. However, NAC can effectively prevent and control the process of oxidative stress development [60,61,62,63,64]. Interestingly, it was found that LEE induced a GSH decrease and GSSG increase, which in turn augmented ROS production. LEE was also able to increase ROS level even in the presence of NAC, and exhibited significant activities by regulating the expression of NAC-induced STAT3, CXCR7/4, and EMT markers. 

In this study, we have demonstrated that LEE can attenuate the invasion and migration potential of breast cancer cells by diverse molecular mechanisms. Importantly, LEE inhibited CXCR7/4 and EMT processes even after stimulation with CXCL12 and overexpression of MnSOD. In addition, LEE demonstrated substantial anticancer efficacy by causing inhibition of the JAK/STAT cascade and stimulating ROS levels. Thus, our findings clearly establish LEE can be potentially developed as a novel agent for breast cancer treatment after successful completion of preclinical and clinical studies in the future.

## 4. Materials and Methods

### 4.1. Reagents

Leelamine (LEE, Figure 1A) was purchased from Cayman Chemical (Ann Arbor, MI, USA). LEE stock solution (10 mM) was prepared in EtOH, storage at −20 °C and finally diluted in cell culture medium for use. Anti-CXCR7 and anti-CXCR4 antibodies were purchased from abcam (Cambridge, UK). Anti-MnSOD, anti-fibronectin, anti-vimentin, anti-MMP-9, anti-MMP-2, anti-N-cadherin, anti-E-cadherin, anti-twist, anti-snail, anti-occludin, and anti-b-actin were purchased from Santa Cruz Biotechnology (Santa Cruz, CA, USA). Anti-phospho-STAT3(Tyr705), anti-STAT3, anti-phospho-JAK1(Tyr1022/1023), anti-JAK1, anti-phospho-JAK2(Tyr1007/1008), and anti-JAK2 were purchased from Cell Signaling Technology (Beverly, MA, USA). Alexa Fluor^®^ 488 donkey anti-rabbit IgG (H+L) antibody and Alexa Fluor^®^ 594 donkey anti-mouse IgG (H+L) antibody was obtained from Life Technologies (Grand Island, NY, USA). The GSH/GSSG-Glo™ Assay kit was purchased from Promega (Madison, WI, USA). 

### 4.2. Cell Lines and Culture Conditions

Human breast cancer cell lines, MDA-MB231, MCF-7, SK-BR-3, and BT-474, were obtained from Korean Cell Line Bank (Seoul, Korea). The normal MCF-10A cells were obtained from American Type Culture Collection (Manassas, VA, USA). The human breast cancer cells were propagated in RPMI-1640 medium supplemented with 10% FBS and 1% antibiotic solution. MCF-10A cells were cultured in DMEM/F-12 medium supplemented with 10% FBS and 1% antibiotic solution. All the cells were maintained at 37 °C in 5% CO_2_ conditions.

### 4.3. MTT Assay

To evaluate the cell viability of LEE-treated cells, the MTT assay was used as described before [65,66]. The cell viability was examined using VARIOSKAN LUX (Thermo Fisher Scientific Inc, Waltham, MA, USA) at 570 nm.

### 4.4. Western Blot Analysis

The protein expression levels were evaluated by Western blot analysis using specific antibodies as described previously [67,68]. The specific bands from the membranes were detected by enhanced chemiluminescence (ECL) kit (EZ-Western Lumi Femto, DOGEN).

### 4.5. RT-PCR

Total RNA was extracted from cells and desired RNA was reverse transcribed and transcripts analyzed as indicated in our previous reports [42,69]. Glyceraldehyde-3-phosphate dehydrogenase (GAPDH) was used as an internal control.

### 4.6. Wound Healing Assay for Cell Migration Observation

The effect on cell migration after LEE treatment was observed by wound healing assay as elaborated previously [44,69]. The cells (6 × 10^5^ cells/well) were seeded on a 6-well plate in a medium without serum, and the wound healing assay was performed. When cell density attained about 80%, wounds were created using a sterile pipette tip. Cells were treated with LEE (1 μM) for 24 h. At 0 h and 24 h the gap of the wound was measured and the graph was plotted.

### 4.7. Boyden Chamber Assay for Cell Invasion Observation

To evaluate the inhibition effects of LEE on cell invasion, the Boyden chamber assay was employed as elaborated earlier [70]. 

### 4.8. Gelatin Zymography

Gelatinolytic activity of MMP-2 and MMP-9 was evaluated by gelatin zymography. The cells (1 × 10^4^ cells/well) were treated with LEE (1 μM) for 24 h. The supernatants were concentrated and prepared in equal amounts for gelatin zymography. The samples were separated on 0.1%-gelatin-containing 10% SDS-PAGE gel. Gels were washed with 2.5% triton X-100 for 1 h, and incubated in zymo-reaction buffer at 37 °C, 5% CO_2_ conditions overnight. Next, gels were stained with Coomassie brilliant blue (7% glacial acetic acid, 40% methanol, 0.25% Coomassie Brilliant Blue R250) then destained with the destaining buffer (10% glacial acetic acid, 10% methanol) until the band was observed [44]. 

### 4.9. Immunocytochemistry

The cells (2 × 10^4^ cells/well) were treated with LEE (1 μM) for 24 h and observed by immunocytochemistry as described earlier [71]. The cells were analyzed using an Olympus FluoView FV1000 confocal microscope (Tokyo, Japan).

### 4.10. Transfection with MnSOD Overexpression

The cells (5 × 10^4^ cells/well) were seeded on a 12-well plate in a medium without serum and transfected with pcDNA3-MnSOD and pcDNA (300 ng) for 24 h by iN-fect™ in vitro Transfection Reagent (iNtRON Biotechnology, Seongnam, Korea). After transfection, cells were treated with LEE (1 μM) for 24 h.

### 4.11. GSH/GSSG Assay

The breast cancer cells (5 × 10^5^ cells/well) were treated with LEE (1 μM) for 12 h. Then GSH/GSSG ratio was evaluated according to the previously described method [72].

### 4.12. ROS Detection Assay

Intracellular production of ROS was measured using cell-permeable fluorescent 2′,7′-dichlorofluorescin diacetate (H2DCF-DA) as described earlier [73]. After treatment with LEE, the cells were harvested and washed with PBS. HDF cells were stained with 10 µM H2DCF-DA at 37 °C for 40 min and analyzed with BD AccuriTM C6 Plus Flow Cytometer (BD Biosciences, Becton-Dickinson, Franklin Lakes, NJ, USA).

### 4.13. Statistical Analysis

All the numerical values have been represented as the mean ± SD. The statistical significance of the data compared with the untreated control was determined using the Student unpaired *t*-test. Significance was set at * *p* < 0.05, ** *p*  <  0.01, and *** *p*  <  0.001.

## Data Availability

The data presented in this study are available on request from the corresponding author.

## Supplementary Materials

The following supporting information can be downloaded at: https://www.mdpi.com/article/10.3390/ijms23179848/s1.
