# Supplementary material for: Potential Application of Leelamine as a Novel Regulator of Chemokine-Induced Epithelial-to-Mesenchymal Transition in Breast Cancer Cells
Source: Int J Mol Sci. 2022 Aug 30;23(17):9848. doi: 10.3390/ijms23179848 (PMC9456465; doi:10.3390/ijms23179848)
Supplement: Supplementary file 1 [file ijms-23-09848-s001.zip › ijms-1824933-supplementary.pdf]

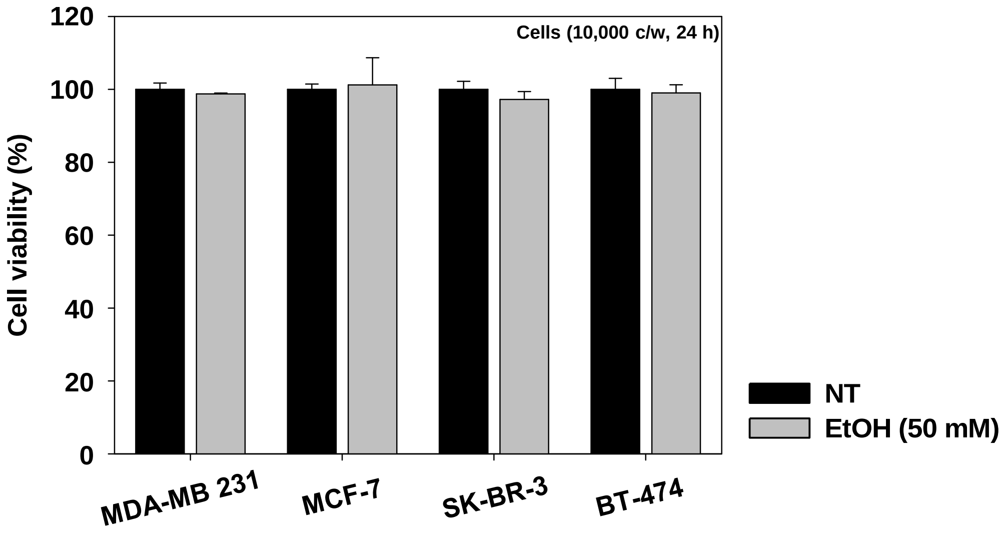

Figure S1. Breast cancer cells were treated with EtOH (50 mM) for 24 h. Then cell viability was measured by MTT assay.

Fig. 1C

Fig. 1D

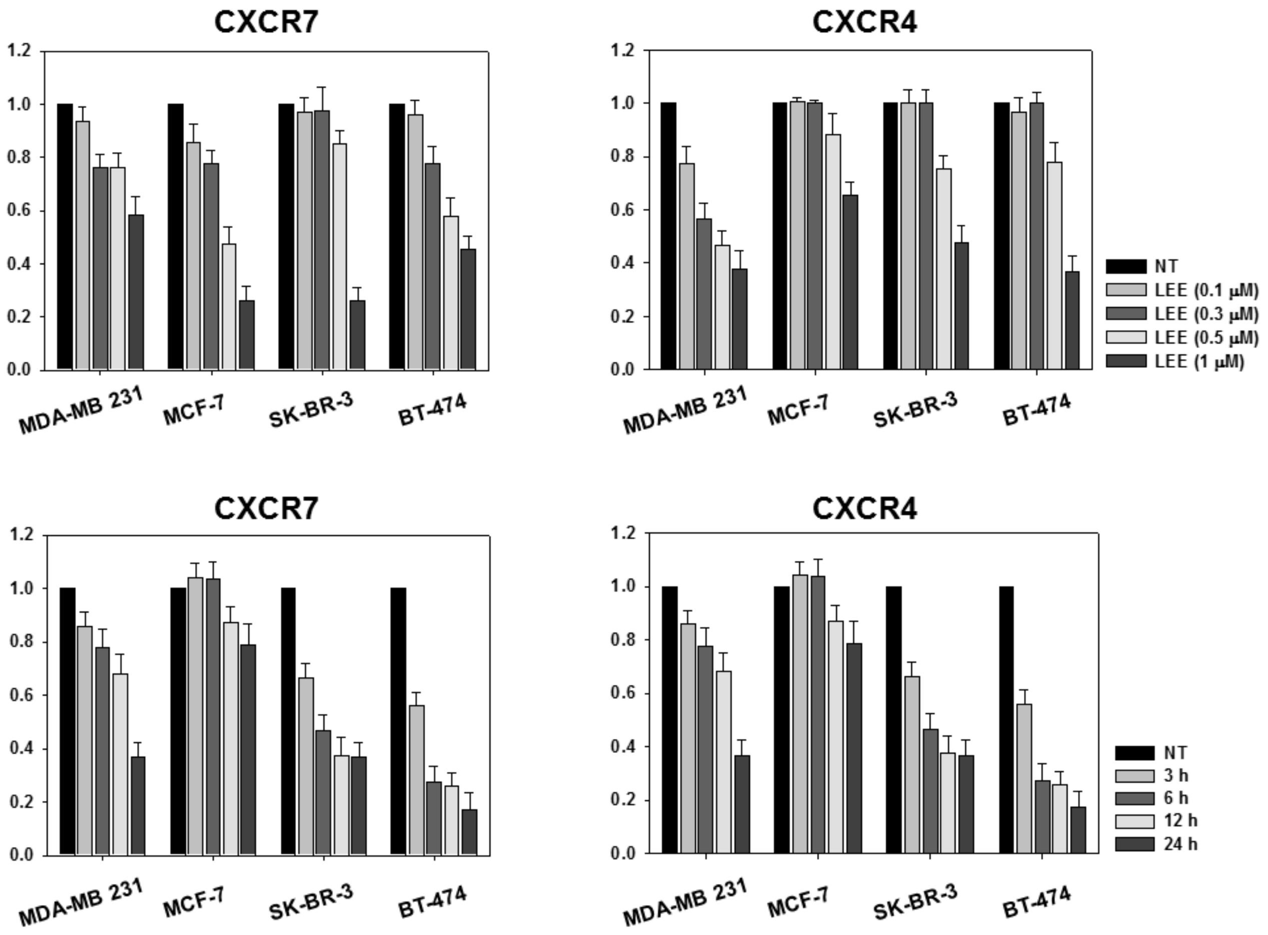

Figure S2. Breast cancer cells were treated by LEE with various indicated concentrations and time conditions. Protein expression levels of CXCR7 and CXC4 were analyzed by Western blot analysis, then stastical analysis were evaluated with NT as the criterion. All the experiments were individually repeated at least thrice.

Fig. 1E

Fig. 1F

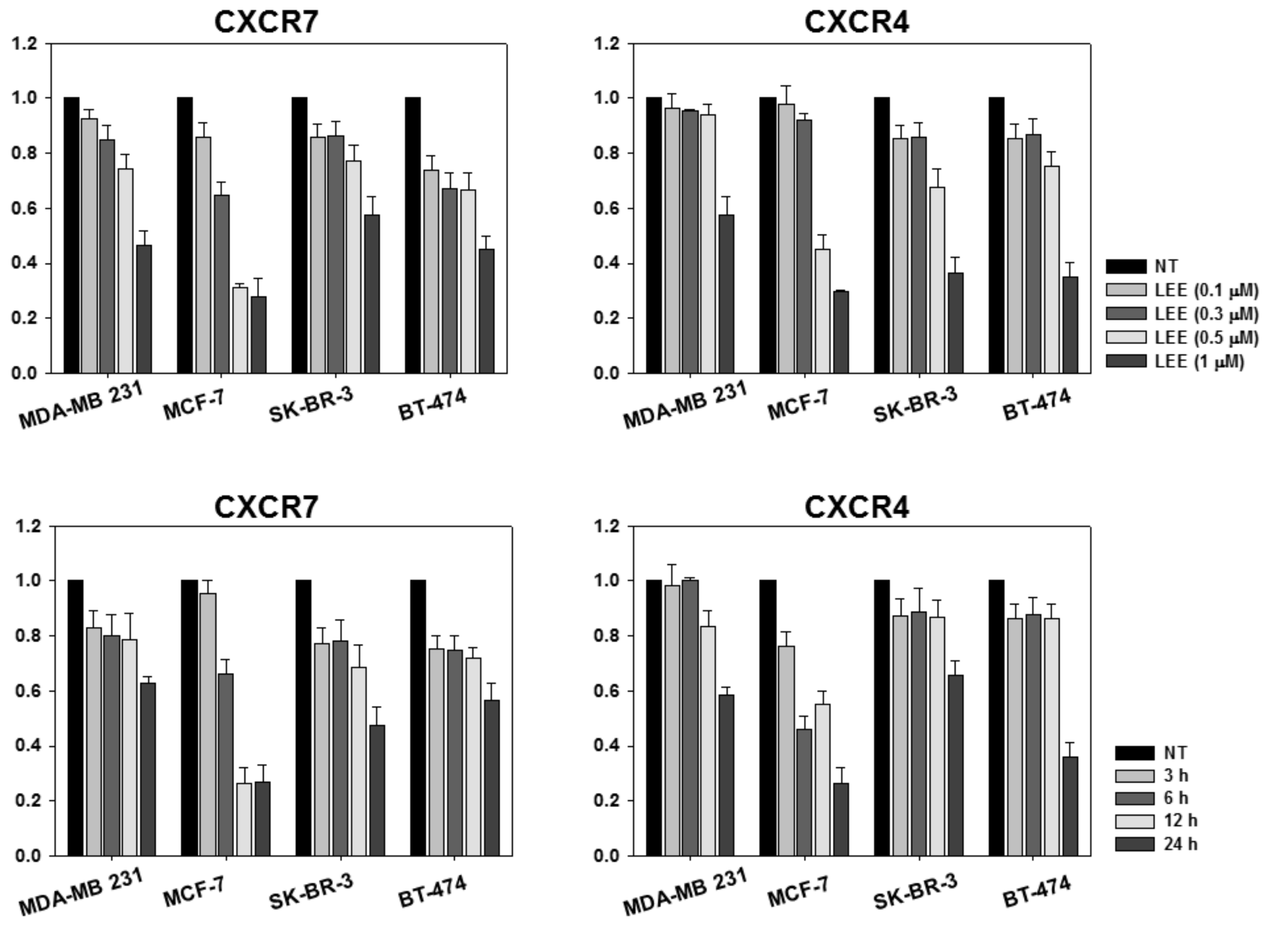

Figure S3. Breast cancer cells were treated by LEE with various indicated concentrations and time conditions. RNA expression levels of CXCR7 and CXC4 were analyzed by Revers transcription PCR, then stastical analysis were evaluated with NT as the criterion. All the experiments were individually repeated at least thrice.

Fig. 2C

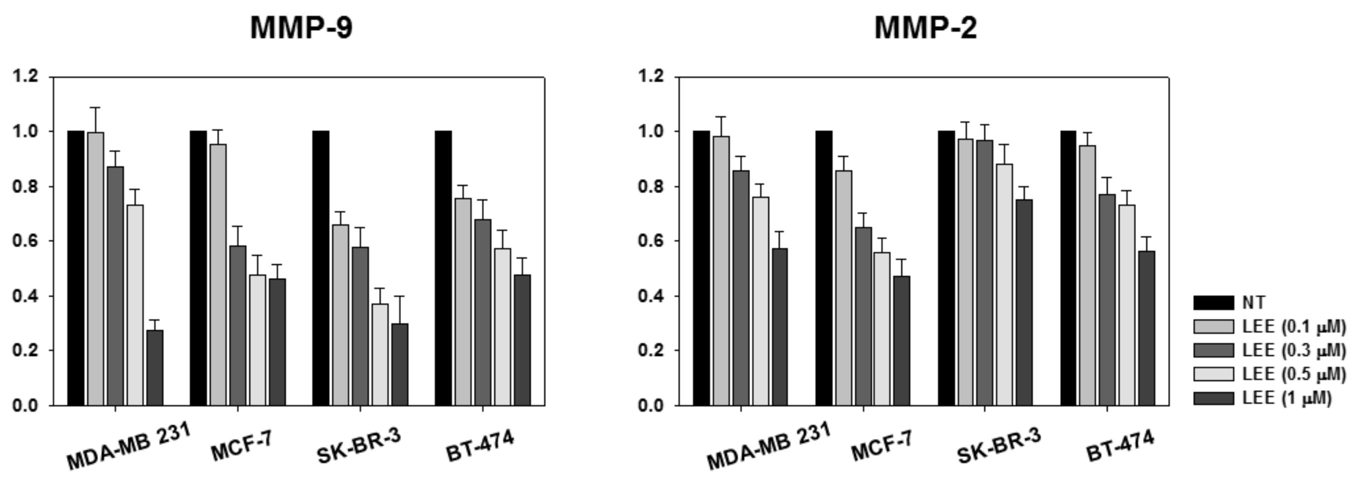

Figure S4. Breast cancer cells were treated by LEE with indicated concentrations conditions. Protein expression levels of MMP-9 and MMP-2 were analyzed by Zymography, then stastical analysis were evaluated with NT as the criterion. All the experiments were individually repeated at least thrice.

Fig. 3A

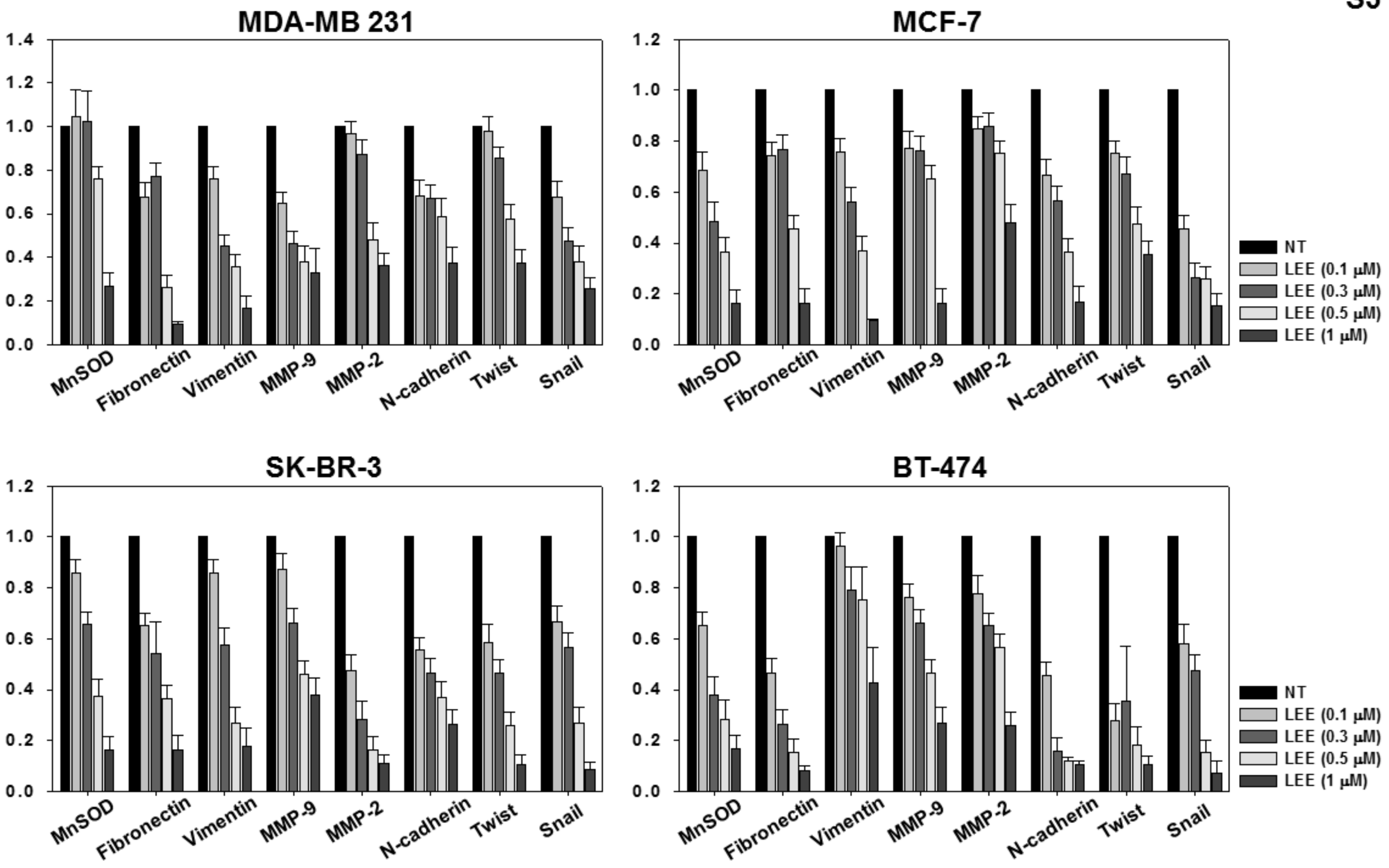

Figure S5. Breast cancer cells were treated by LEE with indicated concentrations conditions. Protein expression levels of MnSOD, Fibronectin, Vimentin, MMP-9, MMP-2, N-cadherin, Twist, and Snail were analyzed by Western blot analysis, then stastical analysis were evaluated with NT as the criterion. All the experiments were individually repeated at least thrice.

Fig. 3B

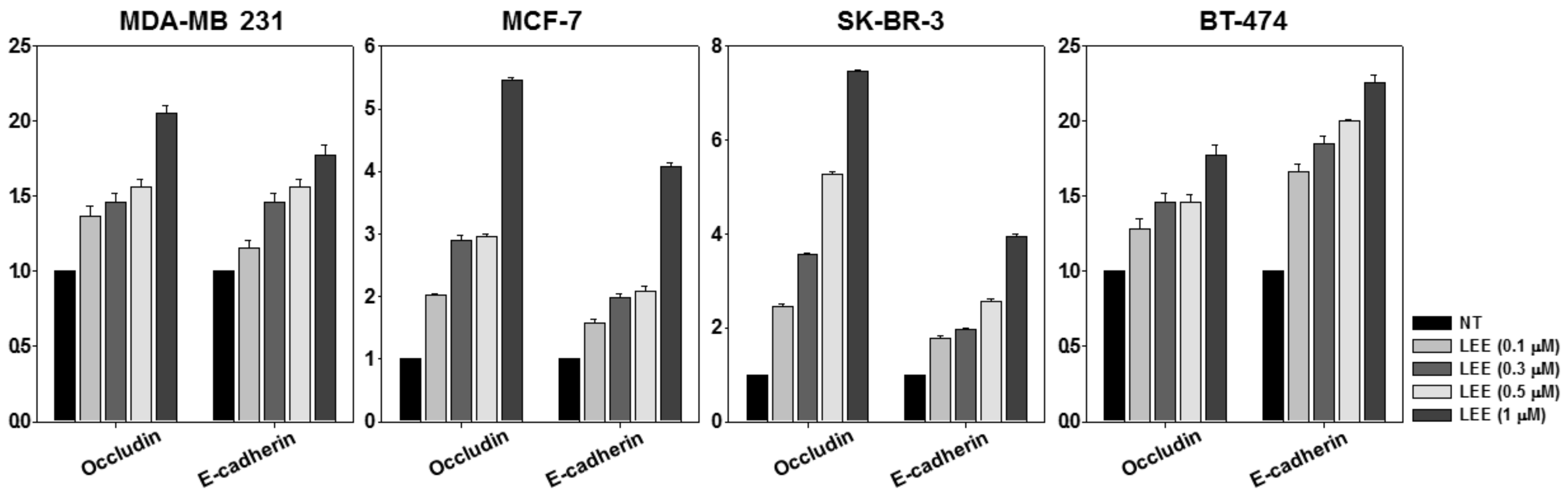

Figure S6. Breast cancer cells were treated by LEE with indicated concentrations conditions. Protein expression levels of Occludin and E-cadherin were analyzed by Western blot analysis, then stastical analysis were evaluated with NT as the criterion. All the experiments were individually repeated at least thrice.

Fig. 4A and B

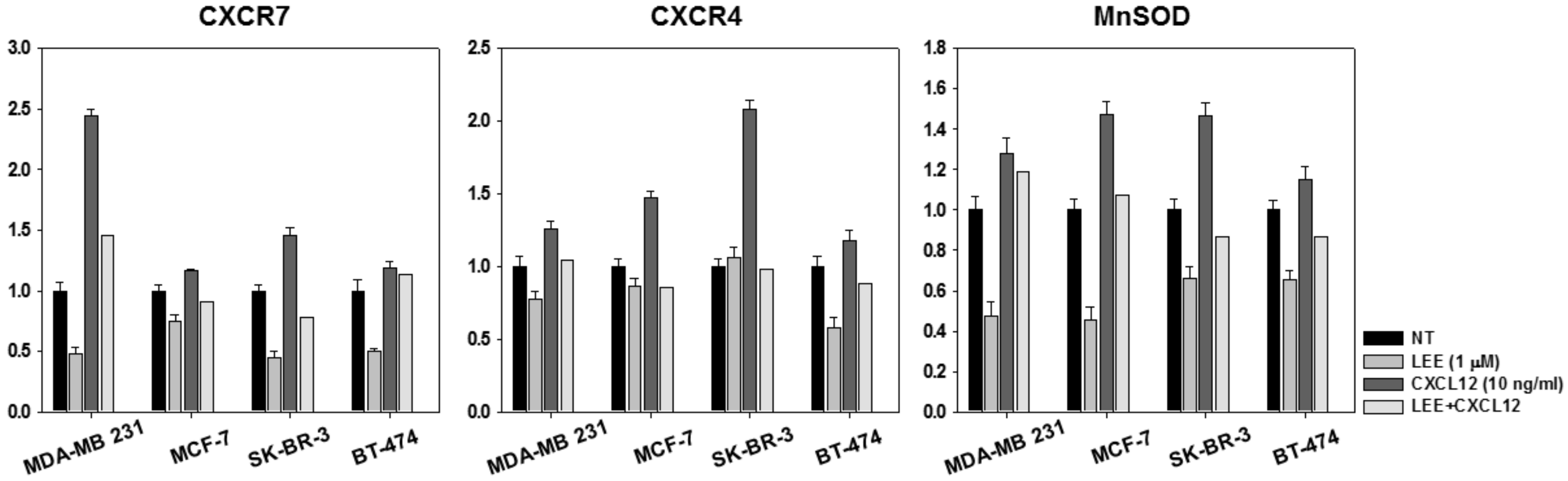

Figure S7. Breast cancer cells were treated by CXCL12 (10 ng/mL) and treated with LEE (1 μM) for 24 h. Protein expression levels of CXCR7, CXCR7, and MnSOD were analyzed by Western blot analysis, then stastical analysis were evaluated with NT as the criterion. All the experiments were individually repeated at least thrice.

Fig. 4C

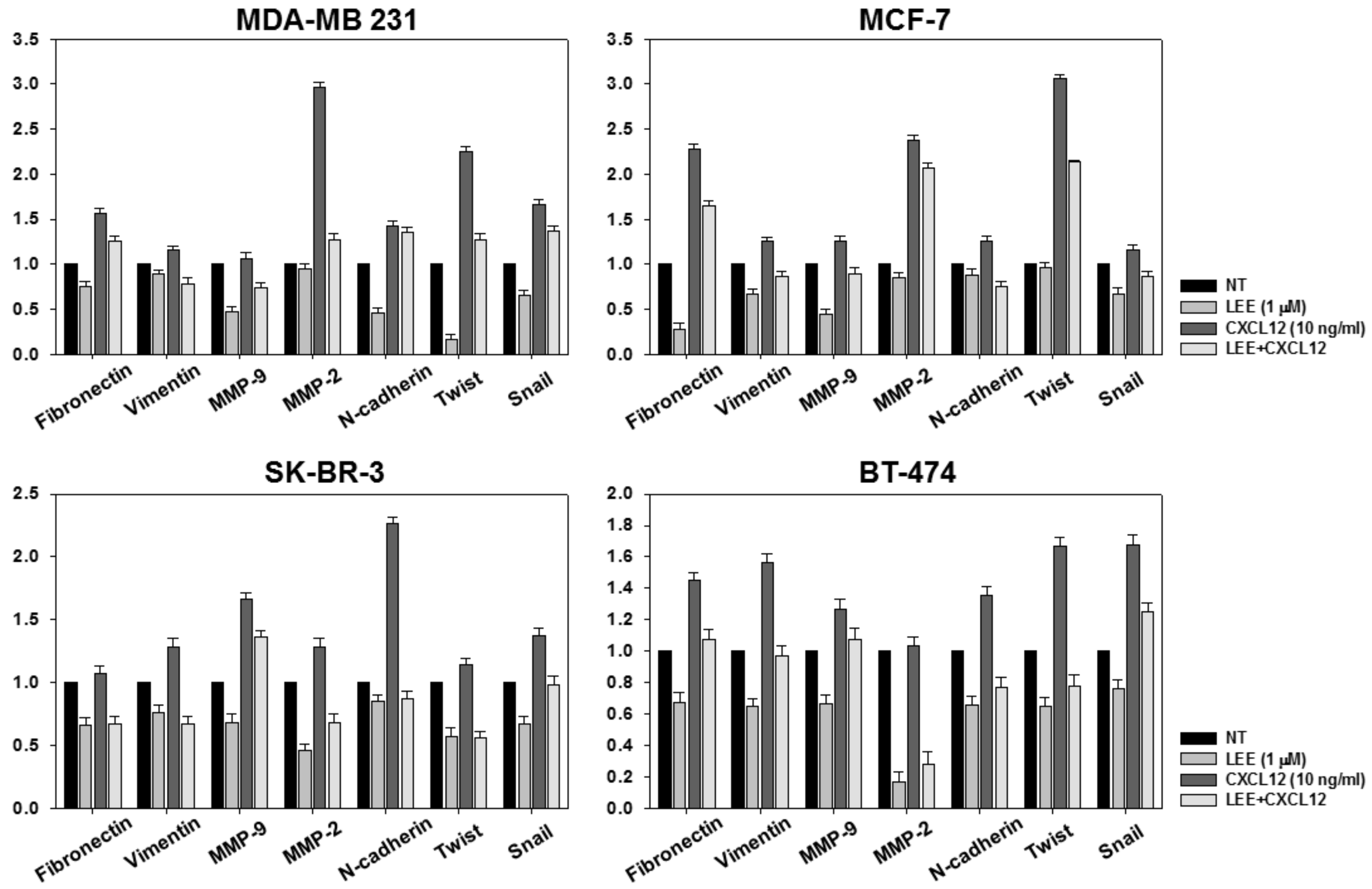

Figure S8. Breast cancer cells were treated by CXCL12 (10 ng/mL) and treated with LEE (1 μM) for 24 h. Protein expression levels of Fibronectin, Vimentin, MMP-9, MMP-2, N-cadherin, Twist, and Snail were analyzed by Western blot analysis, then stastical analysis were evaluated with NT as the criterion. All the experiments were individually repeated at least thrice.

Fig. 4D

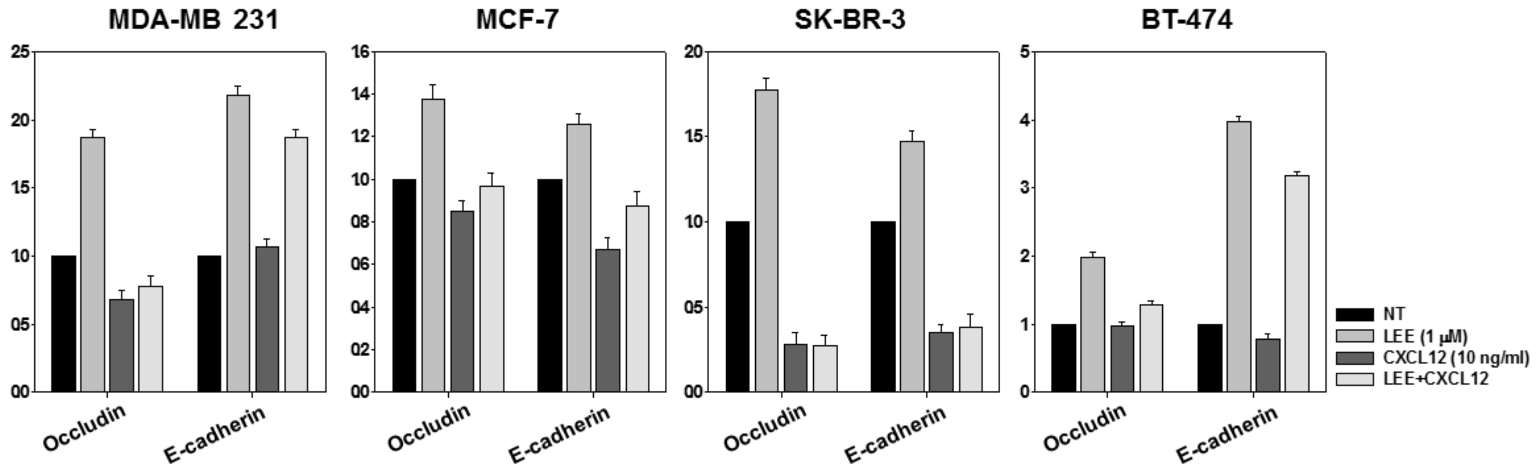

Figure S9. Breast cancer cells were treated by CXCL12 (10 ng/mL) and treated with LEE (1 μM) for 24 h. Protein expression levels of Occludin, and E-cadherin were analyzed by Western blot analysis, then stastical analysis were evaluated with NT as the criterion. All the experiments were individually repeated at least thrice.

Fig. 4E

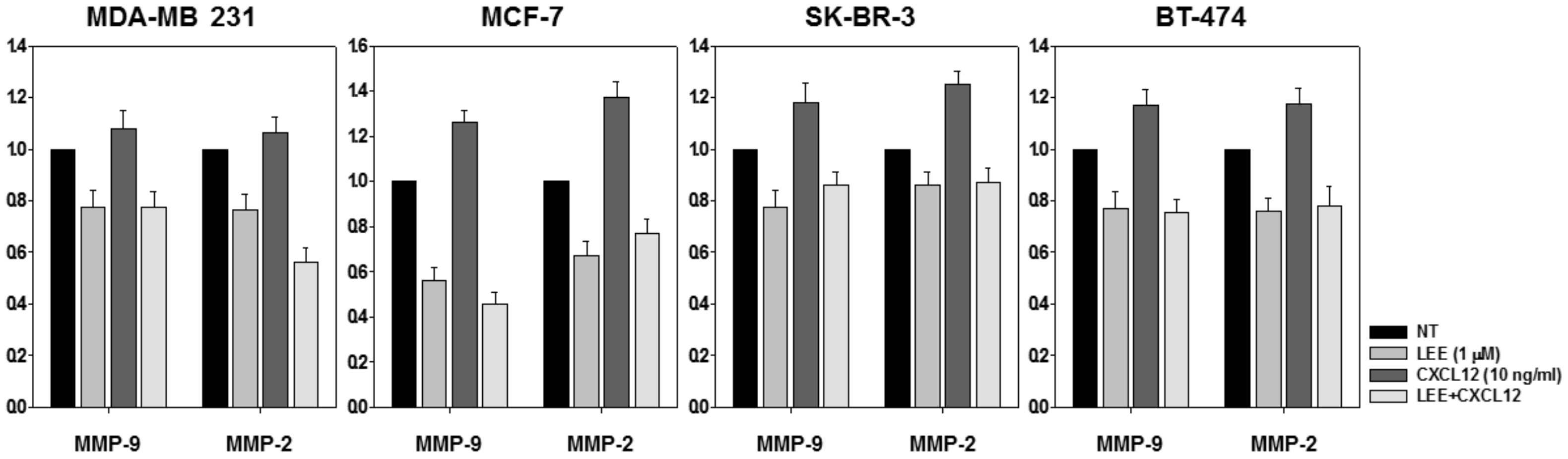

Figure S10. Breast cancer cells were treated by CXCL12 (10 ng/mL) and treated with LEE (1 μM) for 24 h. Protein expression levels of MMP-9 and MMP-2 were analyzed by Zymography, then stastical analysis were evaluated with NT as the criterion. All the experiments were individually repeated at least thrice.

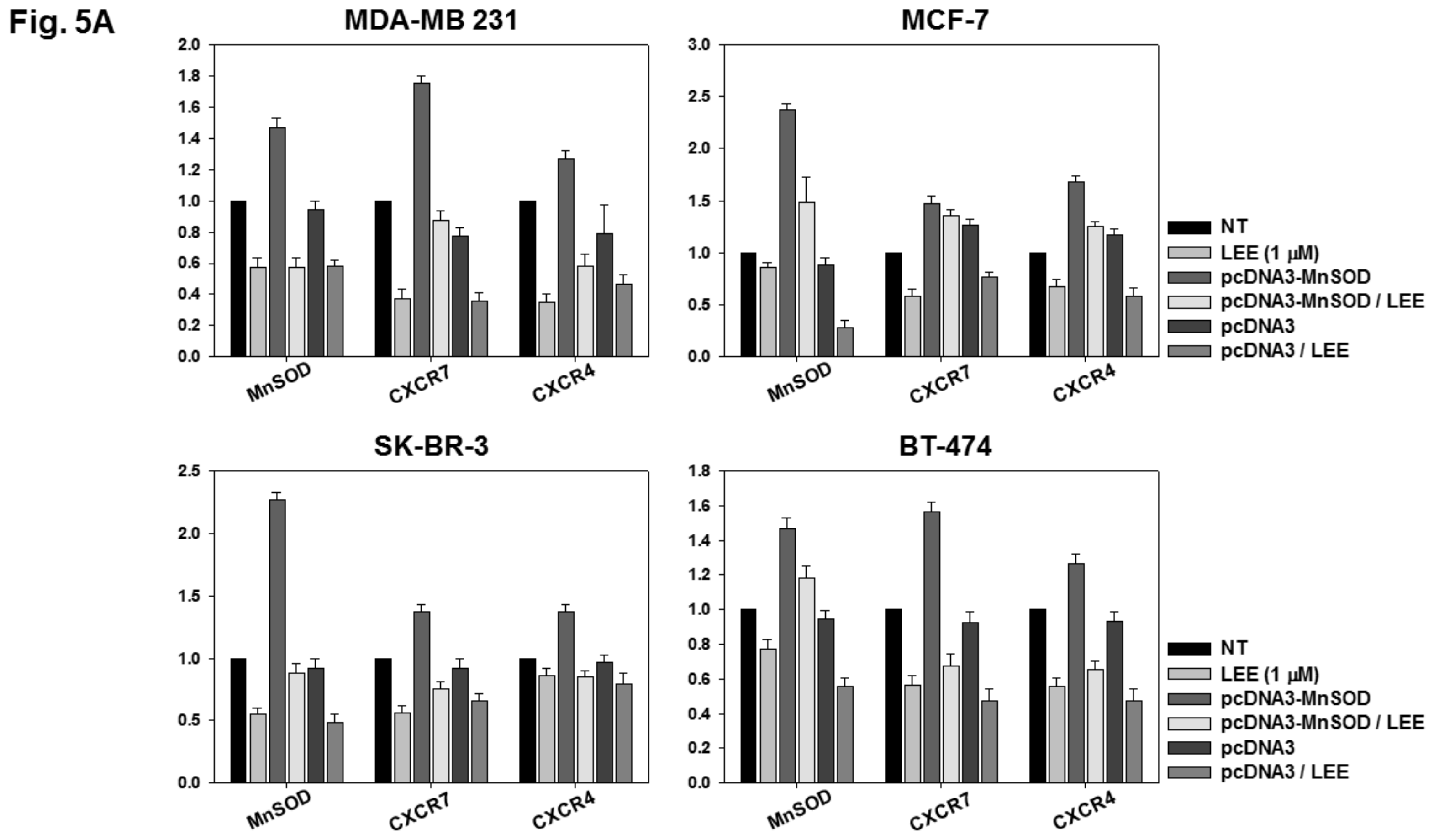

Figure S11. Breast cancer cells were transfected with pcDNA3-MnSOD and pcDNA3 for 24 h, then treated with LEE for 24 h. Protein expression levels of MnSOD, CXCR7, and CXCR4 were analyzed by Western blot analysis, then stastical analysis were evaluated with NT as the criterion. All the experiments were individually repeated at least thrice.

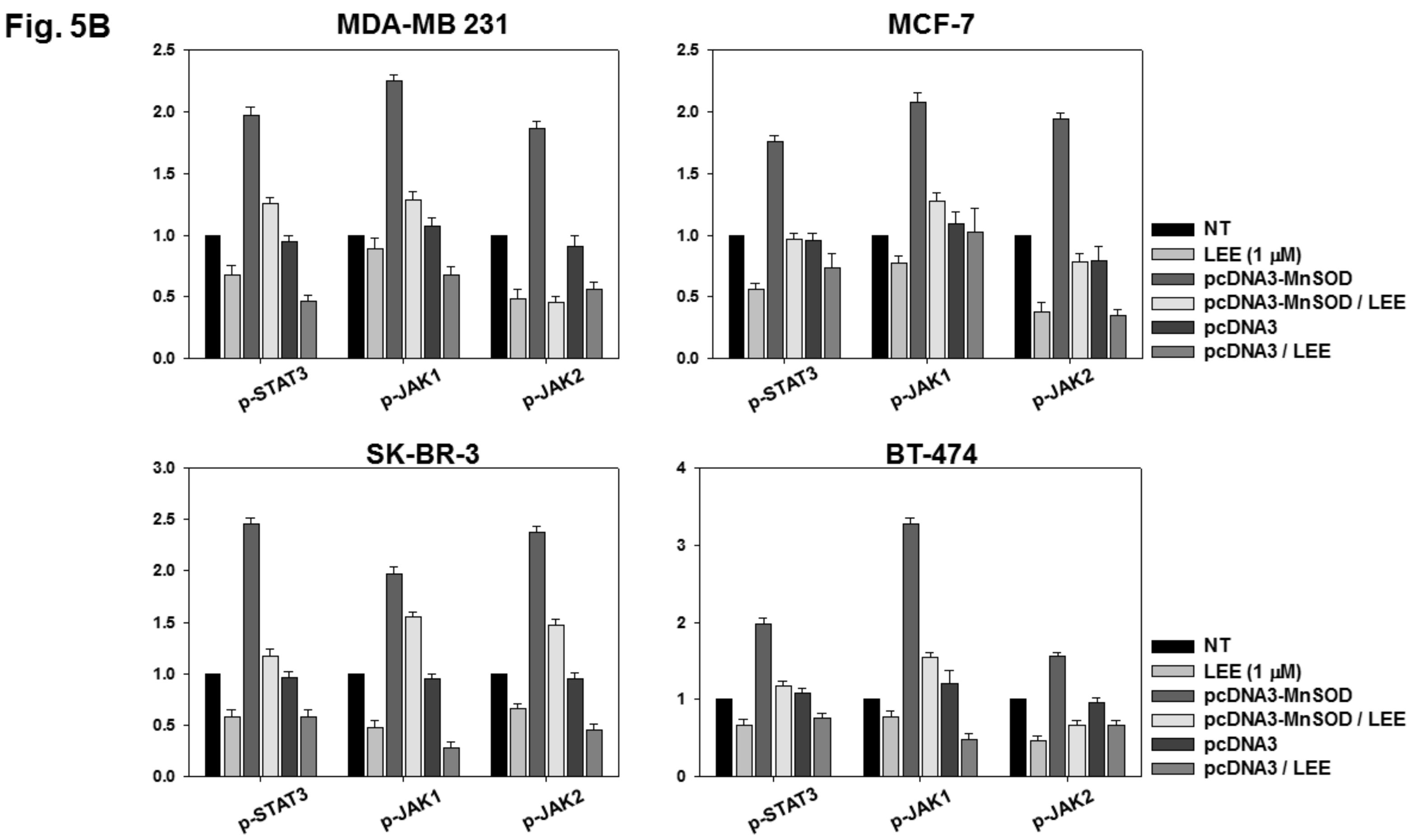

Figure S12. Breast cancer cells were transfected with pcDNA3-MnSOD and pcDNA3 for 24 h, then treated with LEE for 24 h. Protein expression levels of p-STAT3, p-JAK-1, and p-JAK2 were analyzed by Western blot analysis, then stastical analysis were evaluated with NT as the criterion. All the experiments were individually repeated at least thrice.

Fig. 6C

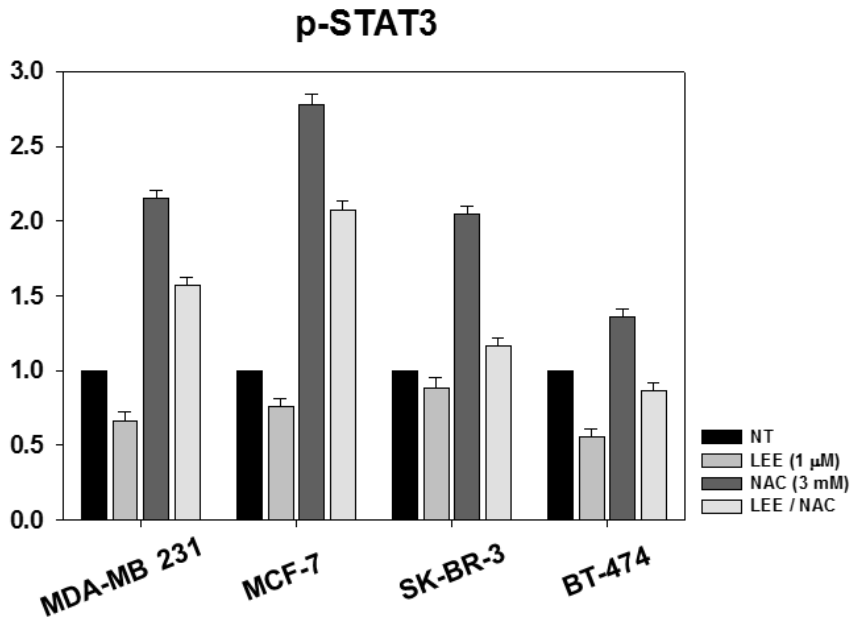

Figure S13. Breast cancer cells were pretreated with LEE and stimulated by NAC for 15 min. Protein expression levels of p-STAT3 was analyzed by Western blot analysis, then stastical analysis were evaluated with NT as the criterion. All the experiments were individually repeated at least thrice.

Fig. 6D

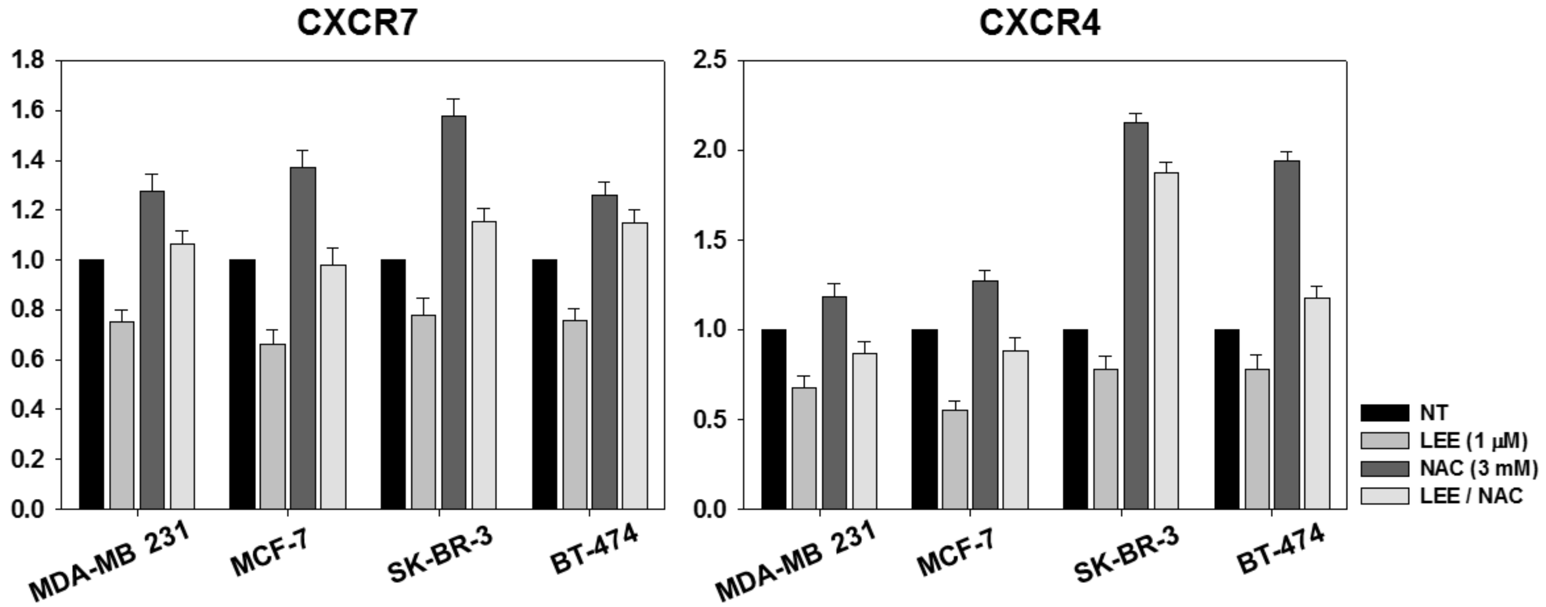

Figure S14. Breast cancer cells were pretreated with LEE and stimulated by NAC for 15 min. Protein expression levels of CXCR7 and CXCR4 were analyzed by Western blot analysis, then stastical analysis were evaluated with NT as the criterion. All the experiments were individually repeated at least thrice.

Fig. 6E

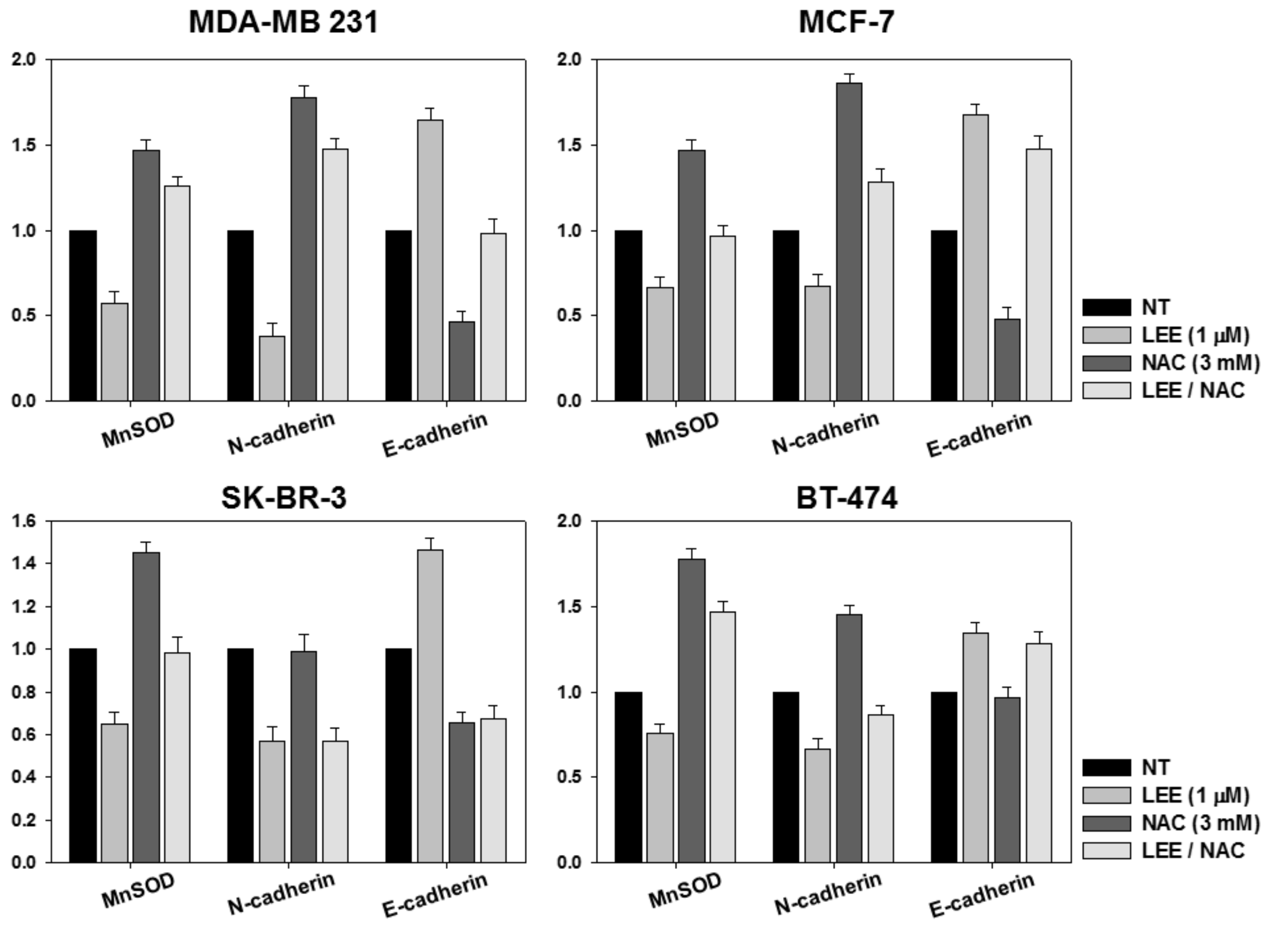

Figure S15. Breast cancer cells were pretreated with LEE and stimulated by NAC for 15 min. Protein expression levels of MnSOD, N-cadherin, and E-cadherin were analyzed by Western blot analysis, then stastical analysis were evaluated with NT as the criterion. All the experiments were individually repeated at least thrice.
